# Single nucleotide polymorphisms in vitamin D binding protein and 25-hydroxylase genes affect vitamin D levels in adolescents of Arab ethnicity in Kuwait

**DOI:** 10.3389/fendo.2023.1257051

**Published:** 2023-10-20

**Authors:** Abdur Rahman, Mohamed Abu-Farha, Arshad Channanath, Maha M. Hammad, Emil Anoop, Betty Chandy, Motasem Melhem, Fahd Al-Mulla, Thangavel Alphonse Thanaraj, Jehad Abubaker

**Affiliations:** ^1^ Department of Food Science and Nutrition, College of Life Sciences, Kuwait University, Kuwait City, Kuwait; ^2^ Department of Biochemistry & Molecular Biology, Dasman Diabetes Institute, Kuwait City, Kuwait; ^3^ Department of Genetics & Bioinformatics, Dasman Diabetes Institute, Kuwait City, Kuwait; ^4^ Special Services Facilities, Dasman Diabetes Institute, Kuwait City, Kuwait

**Keywords:** vitamin D deficiency, single nucleotide polymorphism, *CYP2R1*, vitamin D binding protein, *GC* globular gene, Arab ethnicity

## Abstract

Vitamin D deficiency (VDD) is widespread in the Arab world despite ample sunshine throughout the year. In our previous study, lifestyle and socio-demographic factors could explain only 45% of variability in vitamin D levels in Kuwaiti adolescents, suggesting that genetics might contribute to VDD in this region. Single nucleotide polymorphisms (SNP) in the 25-hydroxylase (*CYP2R1*) and the GC globulin (*GC*) genes have been reported to affect vitamin D levels in various ethnic groups in adults. In this study, we investigated the association of two SNPs from *GC* (rs4588 and rs7041) and three SNPs from *CYP2R1* (rs10741657, rs11023374 and rs12794714) with vitamin D levels and VDD in a nationally representative sample of adolescents of Arab ethnicity from Kuwait. Multivariable linear regression, corrected for age, sex, parental education, governorate, body mass index, and exposure to sun, demonstrated that each of the 5 study variants showed significant associations with plasma 25(OH)D levels in one or more of the additive, recessive, and dominant genetic models - the rs10741657 under all the three models, rs12794714 under both the additive and recessive models, rs7041 under the recessive model; and rs4588 and rs11023374 under the dominant model. Minor alleles at rs4588 (T), rs7041 (A), rs11023374 (C), and rs12794714 (A) led to a decrease in plasma 25(OH)D levels - rs4588:[β (95%CI) = -4.522 (-8.66,-0.38); p=0.033]; rs7041:[β (95%CI) = -6.139 (-11.12,-1.15); p=0.016]; rs11023374:[β (95%CI) = -4.296 (-8.18,-0.40); p=0.031]; and rs12794714:[β (95%CI) = -3.498 (-6.27,-0.72); p=0.014]. Minor allele A at rs10741657 was associated with higher levels of plasma 25(OH)D levels [β (95%CI) = 4.844 (1.62,8.06); p=0.003)] and lower odds of vitamin D deficiency (OR 0.40; p=0.002). These results suggest that the *CYP2R1* and *GC* SNP variants are partly responsible for the high prevalence of VDD in Kuwait. Genotyping these variants may be considered for the prognosis of VDD in Kuwait.

## Introduction

Vitamin D is a fat-soluble vitamin which functions like a steroid hormone in the body and is thus considered as a seco-steroid. Its well-established function in the body is the regulation of blood calcium and phosphate homeostasis and bone metabolism. In addition to this well-established function, vitamin D is also known to be involved in several other physiological functions such as glucose homeostasis, insulin secretion, insulin sensitivity, cardiovascular health, protection from cancers and brain development and function (1–3).

Vitamin D deficiency (VDD) is considered as a global pandemic (4), with over 80% of the population reported to have either insufficient or deficient levels of vitamin D (5, 6). Deficiency is particularly widespread in the Middle East and North Africa (MENA) region. Estimated prevalence rates from various Gulf Cooperation Council (GCC) countries are 83% in the United Arab Emirates, 86% in Bahrain, 85% in Qatar, 81% in Saudi Arabia (7) and 83% in Kuwait (8). Similarly, prevalence of VDD in adolescents in Saudi Arabia and Kuwait was reported to be 96% (7, 9). The very high prevalence of VDD in the MENA region is of particular interest to the medical community as it is a region with abundant sunshine throughout the year. Some of the reported factors associated with VDD in this region are sun avoiding behavior due the high temperature in summer, skin pigmentation, high body mass index (BMI) and dietary factors, particularly the lack of vitamin D supplement and low intake of vitamin D-rich foods (10). In addition, clothing that covers most of the body in both men and women due to cultural and religious reasons are also among the factors associated with high VDD in this region. Our earlier study on a nationally representative sample of adolescents in Kuwait revealed that only 3.9% of adolescents had sufficient levels of vitamin D (9). Major factors associated with VDD in this cohort were age, gender, parental education, time spent outdoor, locality of residence (governorate), BMI and taking vitamin D supplements. However, the best model could explain only 45% of the variability in vitamin D levels (9). This suggests that other factors, particularly genetics, might play a significant part in variation in vitamin D levels (11, 12).

Vitamin D, whether obtained from the diet or endogenously synthesized, must undergo two successive hydroxylation to produce 1,25-dihydroxyvitamin D (1,25(OH)_2_D). This is the active form of the vitamin and functions as a steroid hormone through its binding with nuclear vitamin D receptor (VDR). The first hydroxylation is carried out by hepatic 25-hydroxylase, which converts vitamin D to 25-hydroxyvitamin D (25(OH)D)). Several isoforms of this enzyme exist, of which the lysosomal CYP2R1 is the major 25-hydroxylase (13). It has higher affinity for vitamin D than the other isoforms and hydroxylates both vitamin D_3_ and D_2_ (14). The 25(OH)D) thus formed is the major circulating form of vitamin D and is used as a biomarker for vitamin D status due to its higher concentrations, longer biological half-life and its ability to reflect dietary intakes (6, 14–16).

Being hydrophobic, all the vitamin D metabolites (vitamin D, 25(OH)D and 1,25(OH)_2_D) are circulated bound to proteins, particularly vitamin D binding protein (VDBP). Approximately 99% of the 25(OH)D is bound to protein in the blood, of which 85% is bound to VDBP and 15% to albumin (17, 18). Global studies have reported the association of single nucleotide polymorphisms (SNP) in vitamin D metabolism and transport genes (19–24). Of the many loci associated with vitamin D levels, *CYP2R1*, and *GC* were consistently reported across European and several non-European GWASs; and these loci were also confirmed in GWAS conducted in children/toddlers/new-borns (see [24] **for a review**). In our previous study, we reported on two SNPs in the *CYP2R1* gene (rs10500804 and rs12794714) and one SNP in the *GC* gene (VDBP) (rs1155563) correlating with 25(OH)D levels exclusively in adult Arab population from Kuwait (20). In this study, we considered further most common SNPs from these gene loci namely *GC* (rs4588 and rs7041) and *CYP2R1* genes (rs10741657, rs11023374 and rs12794714) and examined the association between these SNPs and vitamin D levels in adolescent Arab population from Kuwait. We hypothesize that SNPs in the *CYP2R1* and *GC* genes are associated with the circulating levels of 25(OH)2 and thus are, at least partly, contributing to the very high prevalence of vitamin D deficiency in the GCC countries.

## Methods

### Study design

Subject Selection: Adolescents (11-16 years old; N=1416) were selected from public middle schools from all the six Governorates of Kuwait, using stratified multistage cluster random sampling. Ethical approvals were obtained from the Ministry of Health, Kuwait (No: 2015/248), the Health Sciences Centre, Kuwait University (No: DR/EC/2338), and Dasman Diabetes Institute (RA2017-026). Details of the study protocol and sample selection have been previously published (9, 25). Blood samples were collected in February, March, and April 2016. Data on socio-demographic factors and other covariates were collected through self-administered questionnaire completed by the parents and face-to-face interview with adolescents (9). The other covariates included parental level of education, income, type of housing, number of siblings, passive smoking, habitual sun exposure during the previous 3 months, and physical activity. Further, data on dietary intake of vitamin D were collected for 200 of these students, using the Food Frequency Questionnaire for calcium and vitamin D intake in adolescents. A sub-sample of 427 adolescents was randomly selected for the DNA extraction and SNP genotyping. Information on age, sex, parental education, governorate, body mass index, and exposure to sun (the number of times the adolescent walks to school per week) were available for these 427 adolescents. However, information regarding vitamin D supplement was available for only 5 of these 427 adolescents.

### Sample collection and processing

After obtaining written informed consents of the parents and verbal assent of the child, 5 mL of venous blood was collected from each child in heparinized tubes. After centrifugation, plasma was collected for measuring biochemical parameters. The top portion of the pelleted cells, which included mostly white blood cells, was used for DNA extraction. DNA was extracted using the Gentra Puregene® kit (Qiagen, Valencia, CA, USA). Quant-iT™ PicoGreen® dsDNA Assay Kit (Life Technologies, Grand Island, NY, USA) and Epoch Microplate Spectrophotometer (BioTek Instruments) were used to quantify DNA; absorbance values at 260–280 nm were checked for adherence to an optical density range of 1.8–2.1.

### Vitamin D level detection

Detailed protocol that we used for measuring plasma vitamin D levels has been previously published (9). Plasma 25-OH-D was measured in a CAP-accredited laboratory by LC-tandem MS (LC-MS/MS) using the commercially available kit from Chromsystems (Cat. no. 2000/1000/F; Chromsystems Instruments & Chemicals GmbH). Samples were protected from light throughout handling and processing. Vitamin D status was defined using the Endocrine Society cut-off points on the concentrations of 25-OH-D as follows: vitamin D deficiency <50 nmol/L; vitamin D insufficiency 50 to <75 nmol/L; and vitamin D sufficiency ≥75 nmol/L.

### Targeted genotyping

The TaqMan® Genotyping Assay on ABI 7500 Real-Time PCR System from Applied Biosystems (Foster City, CA, USA) was used to perform candidate SNP genotyping. 10 ng of DNA, 5× FIREPol® Master Mix (Solis BioDyne, Estonia), and 1 µl of 20× TaqMan® SNP Genotyping Assay constituted each polymerase chain reaction sample, and thermal cycling conditions were set at 60°C for 1 min and 95°C for 15 min followed by 40 cycles of 95°C for 15 s and 60°C for 1 min. Sanger sequencing, using the BigDye™ Terminator v3.1 Cycle Sequencing on an Applied Biosystems 3730xl DNA Analyzer, was performed for selected cases of homozygotes and heterozygotes to validate genotypes determined by the above techniques.

### Statistical methods

Univariable regression analysis was used to estimate the association between vitamin D levels and genotypes. Logistic regression was used to estimate odds ratio (OR) of vitamin D deficiency in relation to various alleles. Both the logistic regression and the linear regression models were adjusted for the covariates of age, sex, education level of father, education level of mother, the locality of residence of the subjects (governorate), body mass index, and exposure to sun. The covariate of vitamin D supplementation could not be considered as the relevant information was available only for 5 of the 472 genotyped adolescents. Differences in allele frequencies among groups of study subjects were estimated by Chi-square test. Continuous variables, whose data distributions deviated from normality, were presented as median (IQR). Categorical variables were presented as percentages and frequencies. Wilcoxon rank sum test, Pearson’s Chi-squared test and Fisher’s exact test were used appropriately to compare patient characteristics and genotypes between vitamin D sufficient/insufficient group and vitamin D deficient group. The associations were assessed by linear regression under each of the three genetic models (namely, additive, recessive, and dominant mode of inheritance). Statistical analyses were performed using PLINK, version 1.9, and R software, version 4.0.2. A p-value of ≤ 0.05 was considered statistically significant.

## Results

Data were analyzed for 427 adolescents, of which 196(45.9%) were boys and 231(54.1%) were girls. Mean (SD) age was 12.32 (0.86). All the 427 participants were of Arab ethnicity living in Kuwait. Demographic characteristics of the study population are presented in **Table 1**. The demographic characteristics, which showed differences between the two groups of vitamin D status (sufficient/insufficient vs. vitamin D deficient), were considered as confounders, and were used to correct the models for genotype-phenotype associations.

**Table 1:** Demographic characteristics of the study participants.

Characteristic	Vitamin D sufficient/insufficient, N = 77	Vitamin D deficient, N = 350	p-value
**Age in years – Mean (SD)**	12.10 (0.81)	12.37 (0.86)	0.010
**Sex**			<0.001
Male	65 (84%)	131 (37%)	
Female	12 (16%)	219 (63%)	
**Education of father**			0.047
No formal education	0 (0%)	5 (1.4%)	
Completed primary school	6 (7.8%)	51 (15%)	
Completed secondary school	14 (18%)	94 (27%)	
Completed diploma	16 (21%)	71 (21%)	
University degree or above	41 (53%)	124 (36%)	
Unknown	0	5	
**Education of mother**			<0.001
No formal education	2 (2.6%)	5 (1.4%)	
Completed primary school	3 (3.9%)	34 (9.7%)	
Completed secondary school	9 (12%)	92 (26%)	
Completed diploma	11 (14%)	79 (23%)	
University degree or above	52 (68%)	140 (40%)	
**Governorate**			0.026
Ahmadi	32 (42%)	116 (33%)	
Capital	12 (16%)	50 (14%)	
Farawanya	11 (14%)	36 (10%)	
Hawally	15 (19%)	52 (15%)	
Jahra	3 (3.9%)	65 (19%)	
Mubarak-al-Kabeer	4 (5.2%)	31 (8.9%)	

Summary statistics of results from logistic regression analysis to derive the risk of vitamin D deficiency in a case:control analysis with the vitamin D deficient individuals forming the case (n=350) and vitamin D sufficient/insufficient individuals forming the control (n=77) are presented in **Table 2**. The model was corrected for the confounders of age, sex, parental education, governorate, body mass index, and exposure to sun. Odd Ratio (OR) with significant p-value was observed only with rs10741657 (from *CYP2R1*) – the observed values were (OR=0.40; p-value=0.002) indicating the variant as protective.

**Table 2:** Results of logistic regression analysis for odds ratio for the risk of vitamin D deficiency outcome.

Gene	SNP identifier	Physical position	Major allele	Minor allele	MAF in the groups of individuals with vitamin D		χ^2^	Odds Ratio	p-value
					deficiency	sufficiency/ insufficiency			
*GC*	rs4588	4:71752606	G	T	0.185	0.141	1.01	1.94	0.10
*GC*	rs7041	4:71752617	C	A	0.408	0.378	0.29	1.18	0.55
*CYP2R1*	rs11023374	11:14882090	T	C	0.249	0.174	2.39	1.58	0.18
*CYP2R1*	rs12794714	11:14892029	G	A	0.472	0.359	4.00	1.53	0.14
*CYP2R1*	rs10741657	11:14893332	G	A	0.276	0.413	6.90	0.40	0.002

Case-Control study with the vitamin D deficient individuals forming the case (n=350) and vitamin D sufficient/insufficient individuals forming the control (n=77). The model was corrected for the confounders of age, sex, parental education, governorate, body mass index, and exposure to sun.

**Figure 1:**
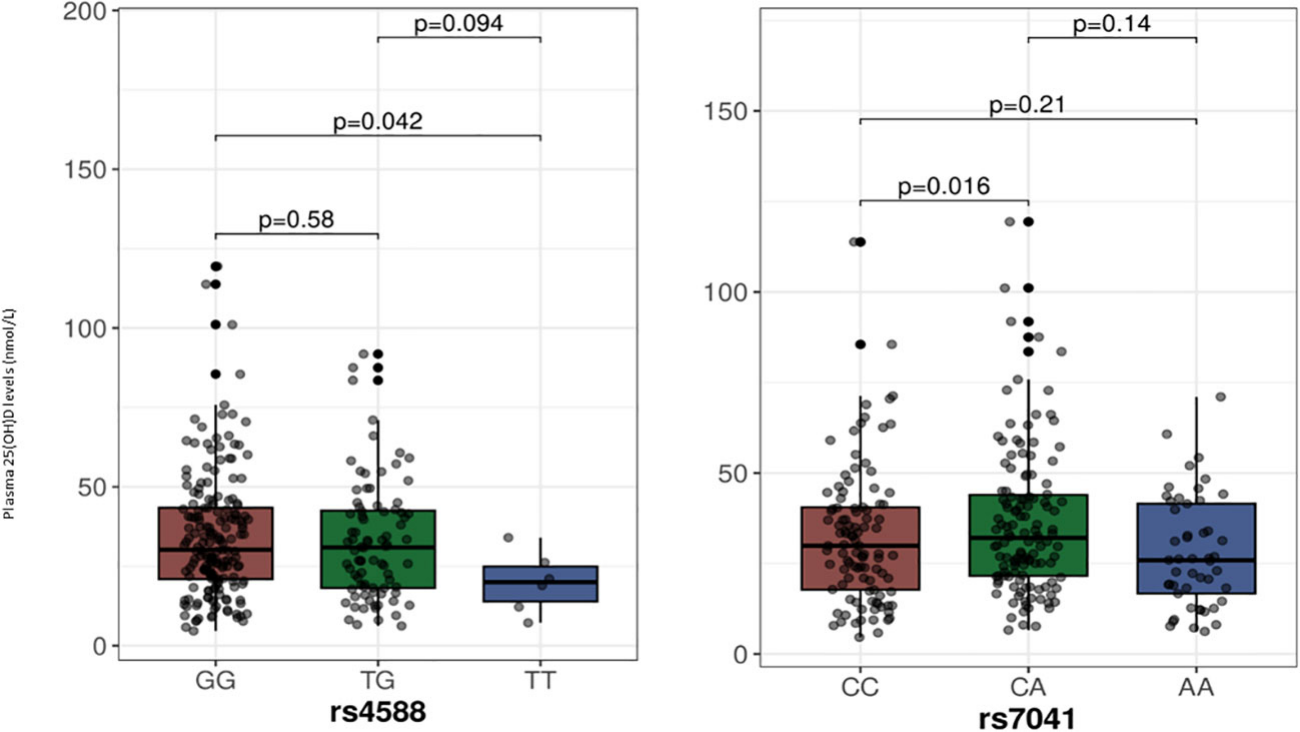
Plasma 25-hydroxyvitamin D levels in relation to the genotypes at SNPs in the *GC* gene. The p-values displayed in the figure correspond to pairwise comparisons between genotypes. The baseline p-value for comparison between the three groups for the rs4588 and rs7041 are 0.13 and 0.04, respectively. We chose to use median with IQR for presentation of 25-hydroxyvitamin D levels as a Kolmogorov-Smirnov test indicated deviation from a normal distribution.

**Figure 2:**
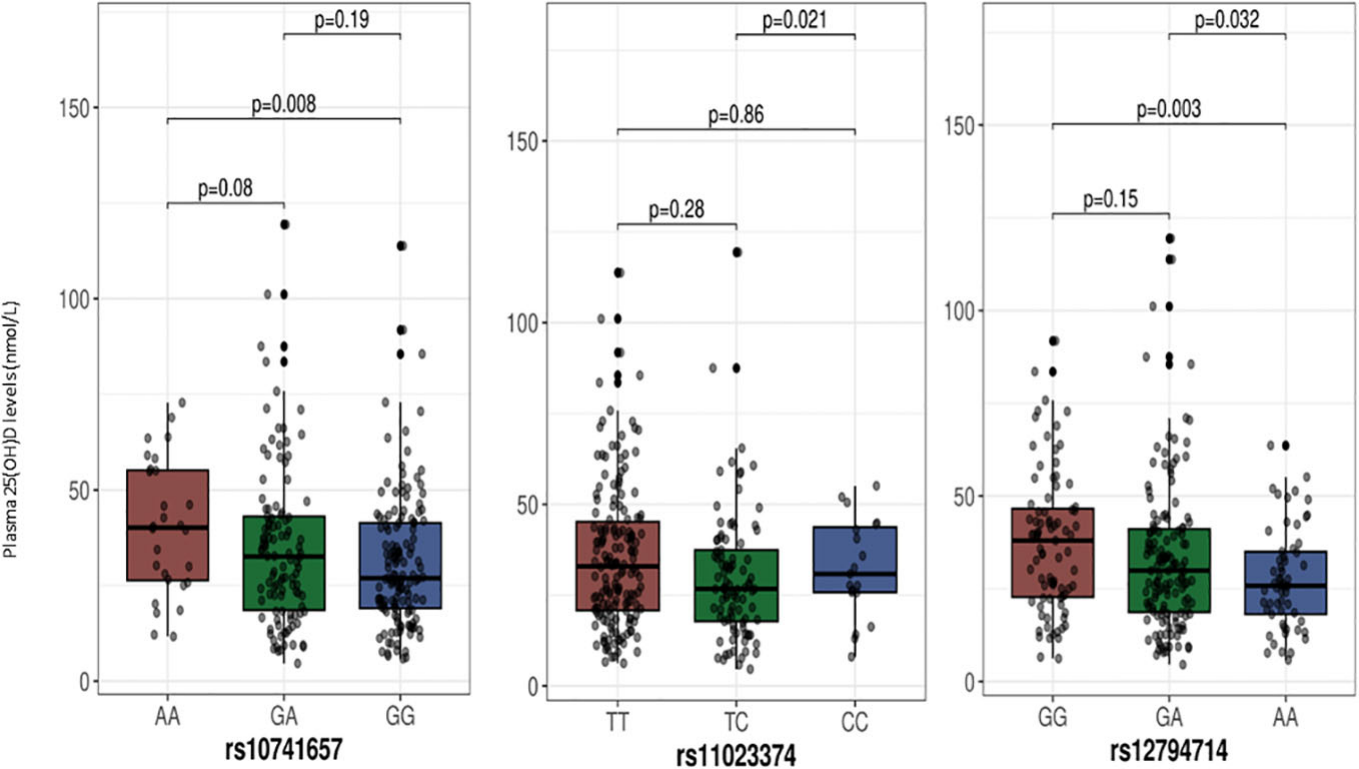
Plasma 25-hydroxyvitamin D levels in relation to the genotypes at SNPs in the CYP2R1 gene. The p-values displayed in the figure correspond to pairwise comparisons between genotypes. The baseline p-value for comparison between the three groups for the rs11023374, rs12794714 and rs10741657 are 0.06, 0.01 and 0. 03, respectively. We chose to use median with IQR for presentation of 25-hydroxyvitamin D levels as a Kolmogorov-Smirnov test indicated deviation from a normal distribution.

The median (IQR) plasma 25(OH)D levels as per the genotypes at the two SNPs from the *GC* gene are shown in **Figure 1**, and among the genotypes at the three SNPs of the *CYP2R1* gene in **Figure 2**. As shown, individuals with the TT genotype homozygous for the minor allele at the rs4588 SNP had significantly lower 25(OH)D levels compared to individuals with the GG genotypes (p= 0.04), but not different from the individuals with the heterozygous GT genotype (p=0.09). On the other hand, 25(OH)D levels in individuals with AA genotypes homozygous for the minor allele at rs7041 SNP were not significantly different from individuals with the other genotypes CC and CA. For the CYP2R1 rs10741657 SNP, individuals with genotype homozygous for the minor allele A (AA genotype) had significantly higher 25(OH)D levels compared to the GG genotype (p=0.008), whereas it was not significantly different from the GA genotype (p=0.19). For the SNP rs11023374, individuals with CC genotype homozygous for the minor allele C had significantly higher 25(OH)D levels compared to the individuals with the heterozygous TC genotype (p=0.02) but not significantly different from the TT genotype (p=0.86). For the rs12794714 SNP, individuals with genotypes homozygous for the minor allele A (AA genotype) had significantly lower 25(OH)D levels compared to the individuals with GA genotype (p=0.03) and GG genotype (p=0.003).

**Figure 3:**
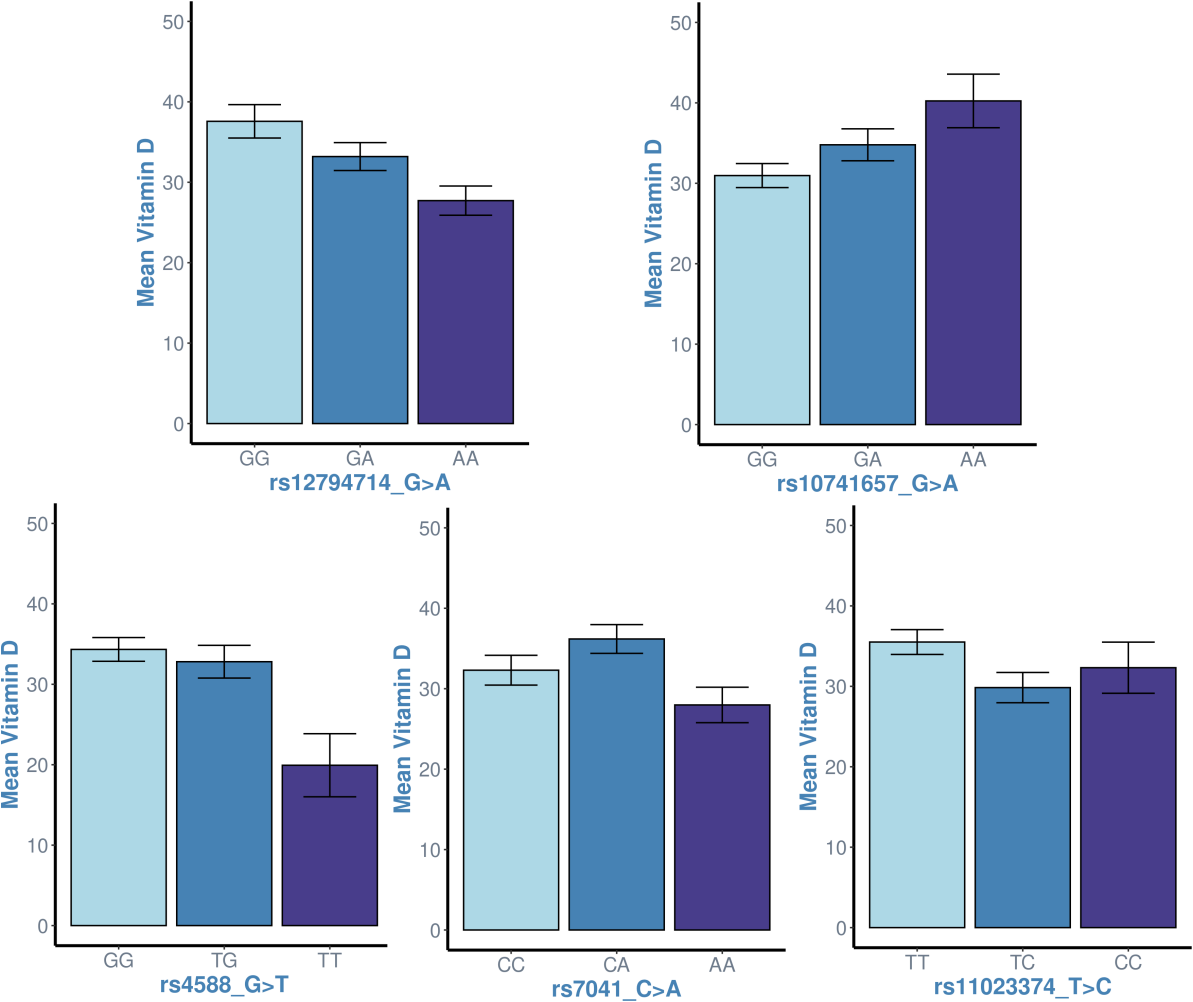
Examining the linearity in the trend between genotypes of increasing minor alleles (genotype homozygous to major allele ➔ heterozygous genotype ➔ genotype homozygous to minor allele) and vitamin D levels at the study variants. x-axis: genotypes; y-axis: mean value of vitamin D levels in individuals with the genotype. The *p*-values associated with linear regression analyses for each SNP are as follows: rs12794714 (*p* = 0.00253), rs10741657 (*p* = 0.0116), rs4588 (*p* = 0.176), rs7041 (*p* = 0.487) and rs11023374 (*p* = 0.06). The plots for rs12794714 and rs10741657 suggest a significant linear association with vitamin D levels (p < 0.05), while the plots for the other three SNPs do not exhibit a linear association.

In an effort to identify the correct genetic model for association tests, we examined the distribution of vitamin D levels across genotypes for each of the five SNPs: rs4588, rs7041, rs11023374, rs12794714 and rs10741657. The trends between increasing minor alleles at the genotypes and vitamin D levels are as presented in **Figure 3**. We noticed a significant linear trend between genotypes of increasing minor alleles and decreased vitamin D levels for rs12794714 (*p* = 0.00253). Conversely, rs10741657 displayed a significant linear trend where additional minor alleles contribute incrementally to elevated vitamin D levels (*p* = 0.0116). These observed linear relationships indicated that the additive genetic mode would be more suited to capture the effects at these two SNPs and provide meaningful insights into both risk and protective associations. The remaining three SNPs (rs4588, rs7041, and rs11023374) did not display a straightforward linear association with vitamin D levels (p > 0.05) and led us to acknowledge that applying additive genetic model to these three SNPs may not capture the potential non-linear effects. Thus, it was felt necessary to uniformly apply all the three genetic models to examine the associations of the study variants with vitamin D levels.

The results of allele-based linear regression analysis (corrected for the confounders of age, sex, parental education, governorate, body mass index, and exposure to sun) for associations between the studied SNPs and the plasma vitamin D levels are presented in **Table 3**, The results point out that each of the five study variants was associated with vitamin D levels with significant p-values in one or more of the three genetic models - the rs10741657 from *CYP2R1* under all the three models, rs12794714 from *CYP2R1* under both the additive and recessive models, the *GC* rs7041 under the recessive model; and the rs4588 from *GC* and the rs11023374 from *CYP2R1* under the dominant model. It is seen that (i) the minor allele T at the rs4588 SNP from *GC* was negatively associated with plasma 25(OH)D levels [β (95%CI) = -4.52 (-8.66, -0.38); p=0.03)] under the dominant model; (ii) the minor allele A at the rs7041 SNP from *GC* was negatively associated with plasma 25(OH)D levels [β (95%CI) = -6.14 (-11.12, -1.15); p=0.03)] under the recessive model; (iii) the minor allele C at the *CYP2R1* rs11023374 SNP was negatively associated with plasma 25(OH)D levels [β (95%CI) = -4.30 (-8.18, -0.40); p=0.03)] under the dominant model; (iv) under the additive model, the minor allele A at *CYP2R1* rs12794714 was negatively associated with plasma 25(OH)D levels [β (95% CI) = -3.5 (-6.27, -0.72); p=0.014); and (v) under the additive model, the minor allele A at *CYP2R1* rs10741657 was positively associated with plasma 25(OH)D level [β (95% CI)= 4.84 (1.62, 8.06); p=0.003)]. As regards the two SNPs, which showed linear trend between increasing minor alleles and change in vitamin D levels (**Figure 3**), the rs10741657 showed significance under all the three models, and rs12794714 showed significance under both the additive and recessive models. The other three SNPs, which did not show linear trend between increasing minor alleles and change in vitamin D levels showed significant associations under recessive or dominant models.

**Table 3:** Multivariable linear regression showing association between GC and CYP2R1 SNPs and plasma vitamin D levels.

	Additive Model			Recessive Model			Dominant Model		
SNP	Beta	95% CI	p-value	Beta	95% CI	p-value	Beta	95% CI	p-value
***GC:*rs4588 (G>T)**	-7.099	-14.27, 0.07	0.053	-12.81	-27.14, 1.53	0.081	-4.522	-8.66, -0.38	0.033
***GC:*rs7041 (C>A)**	-2.457	-5.21, 0.30	0.081	-6.139	-11.12, -1.15	0.016	0.2112	-3.79, 4.21	0.918
***CYP2R1:*rs11023374 (T>C)**	-1.401	-5.37, 2.57	0.489	-1.14	-8.99, 6.71	0.776	-4.296	-8.18, -0.40	0.031
***CYP2R1:*rs12794714 (G>A)**	-3.498	-6.27, -0.72	0.014	-5.177	-9.93, -0.42	0.034	-3.907	-8.10, 0.29	0.069
***CYP2R1:*rs10741657 (G>A)**	4.844	1.62, 8.06	0.003	7.809	1.59, 14.03	0.015	5.392	1.57, 9.21	0.006

The outcome variable, plasma 25(OH)D levels (nmol/L), was used as continuous variable. The model was corrected for the confounders of age, sex, parental education, governorate, body mass index, and exposure to sun.

Statistical significances of the differences in genotype and allele frequencies between the groups of vitamin D sufficient/insufficient and deficient individuals for the studied SNPs are presented in **Table 4**. As seen in the table, the minor allele A at rs10741657 was significantly frequent in vitamin D sufficient/insufficient group (p=0.008), whereas the minor allele A at rs12794714 was significantly frequent in vitamin D deficient group (p=0.042). The AA genotype homozygous for the effect allele at rs10741657 is more frequent in vitamin D sufficient/insufficient group than in deficient group (*p*=0.028).

**Table 4:** Differences in genotype and allele frequencies at the variants between the groups of vitamin D sufficient/insufficient and deficient individuals.

SNP	Vitamin D sufficient/insufficient, N = 77	Vitamin D deficient, N = 350	p-value
rs4588_G>T			
**Genotype**			0.6
**GG**	72%	65%	
**TG**	28%	32%	
**TT**	0%	2.7%	
**Allele**			0.3069
**T**	14%	19%	
**G**	86%	81%	
rs7041_C>A			
Genotype			0.11
AA	8.9%	19%	
CA	58%	43%	
CC	33%	38%	
Allele			0.5959
A	38%	41%	
C	62%	59%	
rs11023374_T>C			
**Genotype**			0.14
**CC**	6.5%	6.9%	
**TC**	22%	36%	
**TT**	72%	57%	
**Allele**			0.1171
**C**	17%	25%	
**T**	83%	75%	
rs12794714_G>A			
**Genotype**			0.12
**AA**	11%	22%	
**GA**	50%	50%	
**GG**	39%	27%	
**Allele**			**0.0419**
**A**	36%	47%	
**G**	64%	53%	
rs10741657_G>A			
Genotype			***0.028***
AA	20%	8.2%	
GA	43%	39%	
GG	37%	53%	
Allele			***0.00814***
A	41%	27%	
G	59%	73%	

## Discussion

In this study we investigated the association between vitamin D levels and SNPs in two genes related to vitamin D metabolism and transport namely the Group-specific Component (*GC*) gene, which codes for VDBP, and the *CYP2R1* gene, which codes for the liver microsomal 25-hydroxylase. Our results can be summarized as follows. Each of the 5 SNPs (from the *GC* and *CYP2R1 genes*) showed significant p-values for associations with plasma 25(OH)D levels, even after corrections for all the confounders, in one or more of the three genetic models (namely additive, recessive, and dominant models) (see **Table 3**). The rs10741657 from *CYP2R1* under all the three models, rs12794714 from *CYP2R1* under both the additive and recessive models, rs7041 from *GC* – under the recessive model; and rs4588 from *GC* and rs11023374 from *CYP2R1* under the dominant model. Minor alleles at rs4588, rs7041, rs11023374, and rs12794714 led to a decrease in plasma 25(OH)D levels while minor allele at rs10741657 led to an increase (see **Table 3**). In accordance with this observation, the frequencies of the minor alleles and genotypes homozygous for minor allele at rs4588, rs7041, rs11023374, and rs12794714 are higher in vitamin D deficient subjects compared to sufficient/insufficient subjects while the frequency of the minor allele and genotype homozygous for minor allele at rs10741657 are higher in vitamin D sufficient/insufficient subjects compared to deficient subjects (see **Table 4**). However, such differences in minor allele frequencies are seen statistically significant only in the following cases. Minor allele T at rs4588 of the *GC* gene is associated with lower plasma 25(OH)D levels. The frequency of this allele is higher (19%) in vitamin D deficient subjects compared to controls (14%) in our study cohort. Similarly, the minor allele A at rs12794714 of the *CYP2R1* is associated with lower levels of plasma 25(OH)D levels. The frequency of this allele is higher (47%) in vitamin D deficient subjects compared to controls (36%) in this cohort. On the other hand, the minor allele A at rs10741657 of the *CYP2R1* is associated with higher levels of plasma 25(OH)D levels and lower odds of vitamin D deficiency (OR 0.40; p=0.002) in this population. The frequency of this allele is lower (28%) in vitamin D deficient subjects compared to controls (41%). These results suggest that SNPs in the genes related to vitamin D transport and metabolism are partly responsible for the very high prevalence of VDD in this population.

We previously reported vitamin D levels and the prevalence of vitamin D deficiency, and the factors associated with it in Kuwait (9). In this population only 3.6% of adolescents had sufficient levels of vitamin D (plasma 25(OH)D levels above 75 nmol/L). Factors that were significantly associated with plasma 25(OH)D levels were age, sex, education level of father, education level of mother, the locality of residence (Governorate), body mass index, vitamin D supplement and the number of times adolescents walk to schools per week. However, the best model could explain only 45% of variation in plasma vitamin D levels. This suggests that other factors are also responsible for such a high prevalence of VDD in this population. Our results, based on genetic models adjusted for age, governorate, parental education, body mass index, and exposure to sun, provide evidence that besides nutritional and environmental factors, genetics also plays a role in determining plasma vitamin D levels. A 23-80% heterogeneity in blood levels of vitamin D has been ascribed to genetic factors (13). Studies reporting varying heritability of vitamin D status by environments, such as season, have suggested that gene-environment interactions (GxE) play a key role (26).

VDBP is encoded by the *GC* gene and is the major carrier of vitamin D metabolites. VDBP has a higher affinity for 25(OH)D. Under normal conditions >99% of 25(OH)D is bound with proteins, mainly VDBP, and only 0.03% is in the unbound form (18). The binding affinity of the VDBP varies with the SNPs in the *GC* gene, with some variants having higher affinity for 25(OH)D than others (27, 28). Different polymorphisms in the *GC* gene produce isotypes of VDBP with different affinities for 25(OH)D, and thus would affect its concentration in the blood (29). As such the presence of these variants may have effects on plasma 25(OH)D concentrations. More than 10 SNPs in the *GC* gene have been studied in relation to plasma 25(OH)D levels (30). Of the several SNPs studied, rs4588 and rs7041 have shown the most consistent association with plasma 25(OH)D levels (11, 31–34). In our study, the rs4588 showed significant association with decreased plasma 25(OH)D levels (p= 0.033) under dominant genetic model, while the rs7041 showed similar significant association with decreased plasma 25(OH)D levels (p= 0.016) under recessive genetic model. Similar to our results, an association between plasma 25(OH)D levels and the *GC* rs7041 SNP was reported in a genome-wide association study on individuals of European ancestry (35). In contrast to our results and those of the above-mentioned global studies, the association of neither the rs4588 and nor the rs7041 with the plasma 25(OH)D levels was established in Chinese population (30). The *GC* rs4588 and the rs7041 are missense variants leading to the amino acid changes of Thr436Met and Asp432Glu, respectively, in the encoded protein and these changes may alter the structure/function of the VDBP.

With regards to the CYP2R1 SNPs, higher vitamin D levels were reported in individuals with the AA genotype at *CYP2R1* rs10741657 SNP in Egyptian populations (36), and in German population (37), indicating that the association of this SNP generalizes to the Arab population as well. A more recent meta-analysis of 16 studies reported that the GG genotype over the AA genotype at this locus was consistently associated with lower levels of vitamin D and with vitamin D deficiency regardless of ethnicity (38). The rs12794714 SNP did not show any association in this meta-analysis while in our studies this variant showed association with decreased vitamin D levels under both the additive and recessive genetic models. All the studies included in this meta-analysis were based on Asian population which could explain the discord between our study, which is based on the Arab population, and these other above-mentioned global studies. The *CYP2R1* SNPs rs12794714 and rs10741657 were also found to be associated with plasma 25(OH)D levels in Han Chinese children (30), in Chinese subjects from Singapore (39), and in Caucasian subjects (40). In particular, the association has been more consistently shown for the rs10741657 in other studies (11, 37). In addition, our study showed association of rs11023374 with decreased vitamin D levels under dominant genetic models. The *CYP2R1* rs12794714 is a synonymous variant leading to no change in amino acid (Ser59Ser) in the encoded protein; but it is in a regulatory region with the feature type of promoter; and the rs10741657 SNP is a non-coding intergenic (*CYP2R1*-*CALCB*) variant. Thus, these two variants. may affect the expression and activity of the 25-hydroxylase enzyme, with consequences for the plasma 25(OH)D levels). The rs11023374 is an intronic variant and its functional consequence has not been deciphered.

In this study, the *GC* SNP rs4588 was associated with lower plasma 25(OH)D levels. The frequency of minor allele T was 19% in vitamin D deficient subjects compared to 14% in the non-deficient (sufficient + insufficient) subjects (p=0.32). On the other hand, the minor allele A frequency of rs12794714, which was associated with lower plasma 25(OH)D levels, was 47% in the deficient vs. 36% in the non-deficient subjects (p=0.042). In contrast, the minor allele A in the *CYP2R1* SNP rs10741657, which was associated with higher plasma 25(OH)D levels was 28% in deficient subjects and 41% in the non-deficient subjects (p=0.009). Together these results indicate that in our population the frequencies of the alleles that are associated with lower vitamin D levels are higher and the frequencies of alleles associated with higher levels are lower, suggesting a strong genetic basis for the prevalence of VDD. The frequencies of these alleles are largely similar to what is reported from the Chinese population (41), and from European population (11).

This work demonstrates that the considered study variants exhibited significant associations with vitamin D levels under different genetic models - the rs10741657 from *CYP2R1* under all the three models, rs12794714 from *CYP2R1* under both the additive and recessive models, the *GC* rs7041 under the recessive model; and the rs4588 from *GC* and the rs11023374 from *CYP2R1* under the dominant model. This observed heterogeneity in the successfully applied genetic models is probably in line with the unique genetic profile of the study cohort. The Arab region is characterized by the cultural factors of close-kin marriage and large families. The practice of consanguineous marriages and the resultant inbreeding has led to accumulation of deleterious recessive alleles in the gene pool. The observed heterogeneity in the successful genetic models has precedence. Our previous genetic association studies on Arabs could identify metabolic risk variants at genome-wide significance mostly under recessive models (42–45) and our recent study illustrated novel association signal between a SNP from SLC17A3 and T1D under recessive model while the association was seen in European population under additive model (46). Most studies test multiple genetic models to explore the biological rationale behind the preference of genetic models.

Given that this study illustrates the genetic influence on vitamin D levels, it would be interesting to explore in future the impact of the reported genetic factors on responses to vitamin D supplementation. An intervention study with genetic assessment, based on the reported *CYP2R1* and *GC* variants, of variability in response to vitamin D supplementation, may help to develop personalized approaches to vitamin D supplementation in the Middle East region.

Our study has several strengths. First, we conducted our study on a nationally representative sample of adolescents in Kuwait. All our subjects were of Arab origin, and thus our results are based on a more homogenous population for genetic studies. Second, our subjects were adolescents in the age range of 11-14 years. The exposure to lifestyle factors like smoking and drinking and other environmental conditions in this adolescent group of the population is minimal and thus the effects of any epigenetic influence on the target variable would be minimal. Third, our data is based on apparently healthy individuals, and thus the effects of any disease on these associations could be ruled out. Many of the previous studies are based on subjects with specific disease conditions like diabetes or cancers. Fourth, we adjusted our statistical analysis for the confounding variables which showed significant association with vitamin D levels in the same cohort, making our results more robust. There are, however, a few limitations in this study. Whereas our original cohort, as reported in our previous study, consisted of 1470 children, the current study considered a sub-cohort of 427 subjects. Although we selected individuals for this sub-cohort randomly as representatives of the original cohort, this lower number might have compromised the robustness of our results. For example, the lack of statistical significance for the odds ratios with 4 of the 5 study SNPs in logistic regression analysis could be due to the smaller sample size. In addition, the cohort had a very high prevalence of vitamin D deficiency. Only 3.6% of the population had sufficient vitamin D. As such in our analysis we had to combine the vitamin D insufficient group with vitamin D sufficient group so that a sizeable control group could be formed in order to have meaningful statistical comparisons with vitamin D deficient group.

In conclusion, we report here that SNPs in the *GC* (VDBP) and *CYP2R1* (25-hydroxylase) genes have significant influence on plasma 25(OH)D levels. This, together with the other social, cultural, and climatic factors, may explain the very high prevalence of vitamin D deficiency in the MENA region. These results warrant that genotyping of these variants be considered for any intervention to deal with the public health problem of VDD in the MENA region. However, these results need to be confirmed on a wider population spectrum.

## Data availability statement

The raw data supporting the conclusions of this article will be made available by the authors, without undue reservation.

## Ethics statement

The studies involving humans were approved by the Ethical Review Committees at Ministry of Health, Kuwait (No: 2015/248), the Health Sciences Centre, Kuwait University (No: DR/EC/2338), and Dasman Diabetes Institute (RA2017-026). The studies were conducted in accordance with the local legislation and institutional requirements. The participants provided their written informed consent to participate in this study.

## Author contributions

AR: Conceptualization, Formal Analysis, Investigation, Resources, Writing – original draft, Writing – review & editing. MA-F: Conceptualization, Data curation, Investigation, Writing – original draft. AC: Data curation, Formal Analysis, Methodology, Software, Writing – original draft. MH: Data curation, Formal Analysis, Methodology, Writing – original draft. EA: Writing – original draft, Data curation, Formal Analysis, Methodology. BC: Writing – original draft, Data curation, Formal Analysis, Methodology. MM: Writing – original draft, Data curation, Formal Analysis, Methodology. FA-M: Resources, Writing – review & editing. TT: Writing – original draft, Writing – review & editing, Conceptualization, Supervision. JA: Conceptualization, Supervision, Writing – review & editing.
